# Antigenic analysis of classical swine fever virus E2 glycoprotein using pig antibodies identifies residues contributing to antigenic variation of the vaccine C-strain and group 2 strains circulating in China

**DOI:** 10.1186/1743-422X-7-378

**Published:** 2010-12-31

**Authors:** Ning Chen, Chao Tong, Dejiang Li, Jing Wan, Xuemei Yuan, Xiaoliang Li, Jinrong Peng, Weihuan Fang

**Affiliations:** 1 Institute of Preventive Veterinary Medicine, Zhejiang Provincial Key Laboratory of Preventive Veterinary Medicine, Zhejiang University, Hangzhou 310029, PR China

## Abstract

**Background:**

Glycoprotein E2, the immunodominant protein of classical swine fever virus (CSFV), can induce neutralizing antibodies and confer protective immunity in pigs. Our previous phylogenetic analysis showed that subgroup 2.1 viruses branched away from subgroup 1.1, the vaccine C-strain lineage, and became dominant in China. The E2 glycoproteins of CSFV C-strain and recent subgroup 2.1 field isolates are genetically different. However, it has not been clearly demonstrated how this diversity affects antigenicity of the protein.

**Results:**

Antigenic variation of glycoprotein E2 was observed not only between CSFV vaccine C-strain and subgroup 2.1 strains, but also among strains of the same subgroup 2.1 as determined by ELISA-based binding assay using pig antisera to the C-strain and a representative subgroup 2.1 strain QZ-07 currently circulating in China. Antigenic incompatibility of E2 proteins markedly reduced neutralization efficiency against heterologous strains. Single amino acid substitutions of D705N, L709P, G713E, N723S, and S779A on C-strain recombinant E2 (rE2) proteins significantly increased heterologous binding to anti-QZ-07 serum, suggesting that these residues may be responsible for the antigenic variation between the C-strain and subgroup 2.1 strains. Notably, a G713E substitution caused the most dramatic enhancement of binding of the variant C-strain rE2 protein to anti-QZ-07 serum. Multiple sequence alignment revealed that the glutamic acid residue at this position is conserved within group 2 strains, while the glycine residue is invariant among the vaccine strains, highlighting the role of the residue at this position as a major determinant of antigenic variation of E2. A variant Simpson's index analysis showed that both codons and amino acids of the residues contributing to antigenic variation have undergone similar diversification.

**Conclusions:**

These results demonstrate that CSFV vaccine C-strain and group 2 strains circulating in China differ in the antigenicity of their E2 glycoproteins. Systematic site-directed mutagenesis of the antigenic units has revealed residues that limit cross-reactivity. Our findings may be useful for the development of serological differential assays and improvement of immunogenicity of novel classical swine fever vaccines.

## Background

Classical swine fever virus (CSFV) is a small, enveloped, positive-stranded RNA virus that causes classical swine fever (CSF), a highly contagious disease of swine and wild boars [1]. CSFV belongs to the genus *Pestivirus *of the family *Flaviviridae*. The genus also includes bovine viral diarrhea virus and border disease virus which are important livestock pathogens [2,3]. CSF viruses can be divided into three major groups with ten subgroups by genetic typing [4]. Recent phylogenetic analyses indicated that there has been a switch in the virus population from the historical group 1 or 3 to the recent group 2 in many European and Asian countries [4-9]. Noteworthy, all live-attenuated vaccine strains used in different countries belong to group 1 [4], including the subgroup 1.1 Chinese lapinized vaccine strain (C-strain) which was derived by serial passage of a virulent strain in rabbits. The C-strain has been used for prophylactic vaccination in China since 1954. Two independent studies also reported that subgroup 2.1 strains recently branched away from the vaccine C-strain and became dominant in China [10,11].

E2 is the major envelope glycoprotein exposed on the surface of the virion. It is essential for virus attachment and entry into the host cells as well as cell tropism [12,13]. This glycoprotein has been implicated as one of the virulence determinants [14,15]. In addition, it can induce neutralizing antibodies and confer protective immunity in pigs [16-21]. The antigenic structure of E2 has been identified using a number of monoclonal antibodies (mAbs). Two independent antigenic units, B/C and A/D (residues 690-800 and 766-865, respectively) have been identified in the N-terminal half of E2 [22,23]. In this context, deletion of the C-terminal half did not affect antibody binding [22-24], and the first six conserved cysteine residues as well as the antigenic motif ^771^LLFD^774 ^are important for the antigenic structure of E2 [22,25].

Genetic diversity of E2 among different groups has been extensively studied [4,10,26-29]. The N-terminal half of E2 is more variable than the C-terminal half [10], suggesting that the antigenic units could be under positive selection apparently due to constant exposure to high immunologic pressure. Different patterns of reactivity with mAbs provided clues of antigenic variation of E2 among different CSFV isolates [11,25,30-33]. A study using neutralizing mAbs to select mAb-resistant mutants showed that, in most cases, single point mutations could lead to complete loss of mAbs binding [22]. Furthermore, amino acid (aa) substitutions at position 710 on the E2 proteins of different strains affected binding and neutralization by a panel of mAbs [34]. Single amino acid exchanges between a group 1 vaccine strain LPC and a group 3 field isolate could totally reverse the mAbs binding pattern [35]. Taken together, variability by one or more amino acids within antigenic units may result in the antigenic variation of E2. To our knowledge, all studies that attempted to resolve antigenic variation of glycoprotein E2 utilized mouse mAbs [11,25,30-35]. No attempt has been made to probe the antigenic variation or group-specific antigenic determinants using anti-CSFV sera from pig, the natural host of CSFV. In addition, little is known about how glycoprotein E2 variation among different CSFV groups and subgroups influences cross-neutralization.

In this study, we raised pig antisera against CSFV vaccine C-strain and a representative subgroup 2.1 strain QZ-07 to assess the extent of antigenic variation within antigenic units of glycoprotein E2. Rabbit polyclonal and mouse monoclonal antibodies were raised against recombinant E2 (rE2) protein from C-strain to evaluate if antigenic variation of E2 results in differences in cross-neutralization. A series of variant C-strain rE2 proteins with single substitutions based on amino acid differences between the C-strain and group 2 isolates were used to define residues involved in antigenic variation of E2.

## Results

### Evaluation of antigenic reactivity of the rE2 proteins expressed in *E. coli*

**Figure 1:**
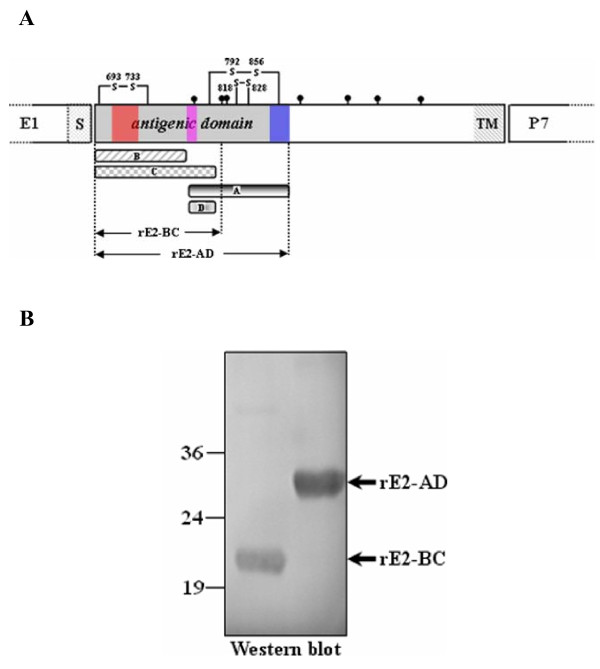
**Generation of prokaryotic-derived recombinant (rE2) proteins**. (A) Schematic presentation of expression of truncated rE2 proteins of CSFV. The antigenic domain of glycoprotein E2 is marked in grey and three antigenic regions identified in this study are marked with different colors. The rE2-BC and rE2-AD proteins expressed in this study are indicated by arrows. The N-linked glycosylation sites (lollipop structures), three disulfide bonds (s-s), the signal sequence (S) and the transmembrane region (TM) are also shown. (B) Antigenic reactivity of the rE2 proteins. The rE2-BC and rE2-AD proteins of CSFV C-strain were expressed in *E. coil*, run through SDS-PAGE and analyzed by Western blot analysis using pig hyperimmune serum against CSFV vaccine C-strain. Molecular weight markers (kDa) are indicated to the left of each panel.

The use of prokaryotic-derived truncated rE2 proteins has been applied in antigen production, antigenic domain identification and epitope mapping [24,36-40]. In this study, two types of truncated rE2 proteins were expressed in *E. coli *Rosetta (DE3) cells (Figure 1A and Table 1). One protein, rE2-BC (aa 690-814), covered the N-terminal 123 residues which are considered to constitute the minimal antigenic domain required for binding to pig anti-CSFV serum [24]. The other protein, rE2-AD (aa 690-865), contained both antigenic units B/C and A/D [22,23]. Western blotting indicated that rE2-BC and rE2-AD proteins of the vaccine C-strain had the molecular weights of 20 and 25 kDa, respectively, and reacted strongly with pig anti-C-strain hyperimmune serum (Figure 1B). Therefore, the prokaryotic-derived rE2 proteins were suitable for use as immunogens to generate polyclonal and monoclonal antibodies as well as for the antibody binding assessments.

**Table 1:** Primers used in PCR amplification of various recombinant E2 proteins

Primer designation^a^	Nucleotide sequence^b^	Target region of E2 protein^c^	CFSV strain amplified	Location in the C-strain genome^d^
C-E2-BC-f	5-AAAGGATCCATGCGCTTAGCCTGCAAGGAAGATTAC	BC unit		2442-2465
C-E2-BC-r	5-AAACTCGAGTCAGAAAGCACTACCG	BC unit		2804-2816
C-E2-AD-f	5-AAGGATCCATGCGGCTAGCCTGCAAG	BC + AD units	Vaccine C-strain	2442-2456
C-E2-AD-r	5-TAGCTCGAGTCAATCTTCATTTTCCAC	BC + AD units		2955-2969
C-E2-f	5-TTTGGATCCGCCACCATGGTATTAAGGGGACAGATCG	Full-size E2		2379-2397
C-E2-r	5-ATTCTCGAGTCAACCAGCGGCGAGTTGTTCTG	Full-size E2		3541-3560
QZ-E2-AD-f	5-AAAGGATCCCGCCTGTCCTGTAAGG	BC + AD units	Subgroup 2.1 Strains	2442-2457
QZ-E2-AD-r	5-TAGCTCGAGGTCTTCTTTTTCTAC	BC + AD units		2955-2969

^a^f, forward; r, reverse.

^b^Underline represents the restriction enzyme digestion sites used for cloning.

^c^See Figure 1 for the various regions of E2 protein.

^d^Locations are derived from the genome of classical swine fever virus C-strain (GenBank accession no. HM175885).

### Reactivity of pig anti-CSFV sera with different rE2-AD proteins

**Figure 2:**
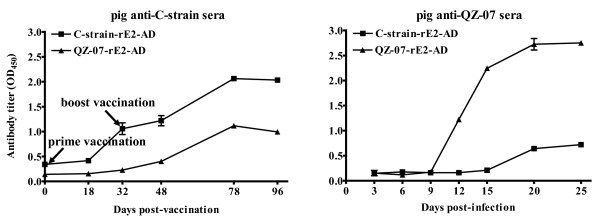
**Reactivity of pig anti-CSFV sera with rE2-AD proteins of CSFV C-strain and strain QZ-07**. The reactivity of rE2-AD proteins of C-strain and strain QZ-07 were cross-examined by indirect ELISA. The antisera were obtained from pigs after immunization with the C-strain or infection with strain QZ-07.

**Figure 3:**
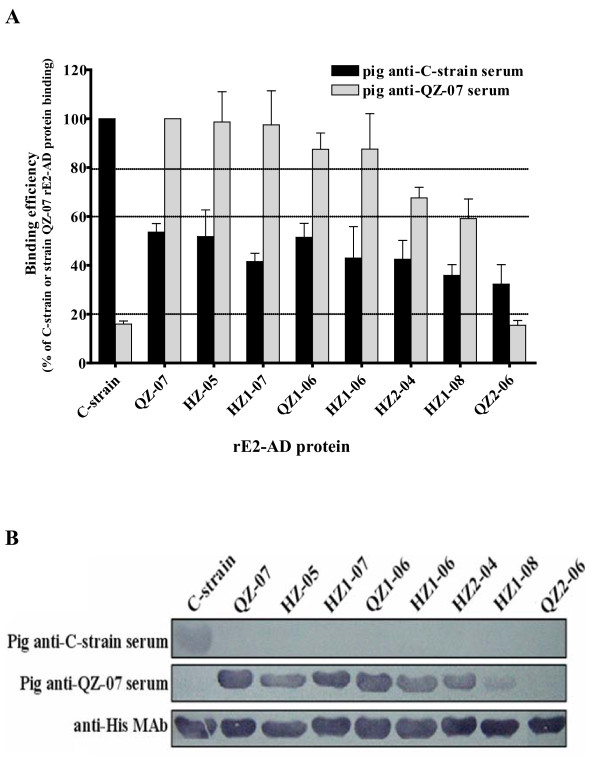
**Binding efficiency of pig anti-CSFV sera with different rE2-AD proteins**. (A) Binding of the rE2-AD proteins from the C-strain and eight subgroup 2.1 strains to pig antisera collected at 78 days post immunization with the C-strain or 25 days post infection with strain QZ-07, respectively. For each of the rE2-AD proteins, the binding efficiency was determined by normalizing to anti-His-tag binding first, and then to C-strain protein or strain QZ-07 rE2-AD protein binding for anti-C-strain or anti-QZ-07 sera, respectively. Thus homologous binding efficiency was set at 100%. Error bars represent standard deviation from three separate experiments. (B) Western blots of rE2-AD proteins using pig anti-C-strain serum, pig anti-QZ-07 serum and mouse monoclonal anti-His-tag antibody.

To assess the antigenic variation of E2 between the subgroup 1.1 C-strain and subgroup 2.1 field isolates, the respective rE2-AD proteins were cross-examined by ELISA with antisera collected from pigs at different time points after immunization with the vaccine C-strain or infection with strain QZ-07 (representing subgroup 2.1). Figure 2 shows that each antiserum reacted much more strongly with rE2-AD protein of the homologous strain (used to prepare the serum) than that of the heterologous strain. Figure 3 further compares binding efficiency of anti-C-strain and anti-QZ-07 sera (collected at 78 days post immunization with the C-strain and 25 days post infection with strain QZ-07, respectively) to rE2-AD proteins derived from C-strain and 8 subgroup 2.1 strains. The homologous binding efficiency was set at 100%. The anti-C-strain serum exhibited significantly low efficiency of binding to subgroup 2.1 rE2-AD proteins (below 60% efficiency). Binding of anti-Q7-07 serum to the C-strain rE2-AD protein was even more inefficient (below 20% efficiency), and the band was barely visible on the blot. Binding of anti-QZ-07 serum to heterologous subgroup 2.1 proteins was varied. While binding with the majority of these proteins was strong (above 80% efficiency), the efficiency of binding with rE2-AD proteins of HZ1-08 and QZ2-06 was below 60% efficiency resulting in faint bands on the blot.

### Neutralization of different viruses by anti-CSFV sera or E2-specific antibodies

**Figure 4:**
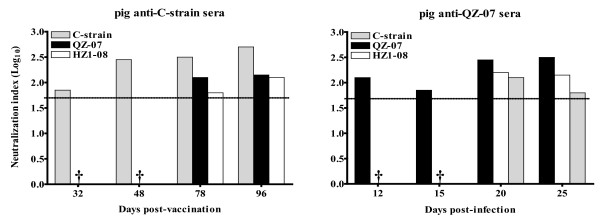
**Neutralization of different CSFV strains by pig anti-CSFV sera**. Virus-specific neutralizing antibodies were cross-examined by neutralization assay with antisera collected from pigs as indicated in Figure 2 legend. Two-fold serial dilutions of the different heat-inactivated sera were mixed with equal volumes of 100 TCID_50 _of the viruses, incubated at 37°C for 1 h and subsequently transferred to confluent monolayers of ST cells in 96-well plates. The starting dilution of each serum was 1:50. At 72 hours post-infection, the cells were fixed and stained for the presence of E2 glycoprotein by immunofluorescence assay. Neutralization index (NI) is the log_10 _of the antibody dilution factor (reciprocal of dilution) when 50% of the wells are protected from infection. Since the starting dilution factor was 50, the NI value of 1.7 is the detection threshold of our neutralization assay. Neutralization indices below 1.7 are indicated as "**†"**.

A two-way neutralization analysis using the pig anti-CSFV sera revealed that heterologous neutralization was less effective, especially with sera collected at the early days following vaccination or infection (Figure 4). Interestingly, neutralization efficiency also differed between subgroup 2.1 strains QZ-07 and HZ1-08. Since strain variation influences the ability of antisera to neutralize heterologous viruses, and inefficient binding of antisera to heterologous rE2-AD proteins was also observed (Figure 3), we sought to determine whether variation of glycoprotein E2 affects CSFV cross-neutralization. Thus, we raised a rabbit antiserum (polyclonal antibodies) and three monoclonal antibodies (mAbs) against C-strain rE2-AD protein. The rabbit antiserum neutralized the QZ-07 virus less efficiently (log_10 _1.8) than the C-strain (log_10 _2.1). Furthermore, substitution of cysteine residues in the antigenic unit B/C with serine residues abolished the reactivity of mAbs 1E7 and 6B8 to E2. However, such mutagenesis did not affect the reactivity of mAb 2B6 (Table 2). These results indicate that these cysteine residues are involved in the structural conformation of E2 [22,23] and that mAbs 1E7 and 6B8 bind to conformational epitopes. In addition, mAb 2B6 only bound to C-strain although its neutralization efficiency was low. The conformational mAbs 1E7 and 6B8 bound to both the C-strain and heterologous subgroup 2.1 viruses but they were less efficient at binding to and neutralizing subgroup 2.1 strains (Table 2). Collectively, these data indicate that strain and glycoprotein E2 variation affect CSFV cross-neutralization.

**Table 2:** Characteristics of three monoclonal antibodies against recombinant E2-AD protein of the vaccine C-strain

			Western blot^a ^(rE2-AD protein)			IFA^b ^(virus infected cells)			Antibody binding/neutralization efficiency^c^		
			
mAb	Isotype	Epitope	C-strain	QZ-07	HZ1-08	C-strain	QZ-07	HZ1-08	C-strain	QZ-07	HZ1-08
1E7	IgG1	Conformational epitope In antigenic unit B/C	-	-	-	+	+	+	5.3/3.35	3.2/<1.7	2.9/<1.7
2B6	IgG2b	Linear epitope at position 1-110 aa	+	±	±	+	-	-	4.4/<1.7	0/0	0/0
6B8	IgG2b	Conformational epitope in antigenic unit B/C	-	-	-	+	+	+	5.6/4.85	4.4/<1.7	4.1/<1.7

^a^Values represent binding of mAbs to denatured prokaryotic-derived rE2 proteins as detected by Western blotting: "+"= strong reactivity; "±"= weak reactivity; "-"= no reactivity detected.

^b^Values represent binding of mAbs to native viral E2 proteins detected by immunofluorescence assay: "+"= fluorescent signal detected; "-"= no signal detected.

^c^IFA and neutralization assay were performed by serial dilutions of mAbs to assess the antibody binding and neutralization efficiency with different CSFV strains.

### Identification of amino acid residues associated with antigenic variation of E2

To determine the amino acid residues responsible for the observed antigenic variation, E2 sequences of 108 CSFV strains representative of each group were obtained from GenBank and aligned. Twenty major variable residues were identified within the antigenic units. Table 3 shows the variability of these residues between vaccine strains and representative group 2 strains.

**Table 3:** Summary of variable sites in the glycoprotein E2 between CSFV vaccine strains and representative group 2 strains

CSFV vaccine strains and representative group 2 strains^a^				**Antigenic unit**^b^																			
				
				Antigenic unit B/C											Overlapping region					Antigenic unit A/D			
**Strain**	**Country**	**Subgroup**	**GenBank accession no.**	**692**	**705**	**706**	**709**	**713**	**723**	**725**	**729**	**736**	**738**	**745**	**777**	**779**	**780**	**788**	**789**	**847**	**854**	**860**	**863**

C-strain	China	1.1	HM175885	A	D	E	L	G	N	D	N	S	V	T	N	S	T	R	S	D	M	T	N
LOM	Korea	1.1	EU789580	A	N	E	L	G	N	D	N	I	V	T	N	S	T	G	F	D	M	T	N
GPE	Japan	1.1	D49533	A	N	E	L	G	N	D	N	I	V	T	N	S	T	G	F	D	M	T	N
Riems	Germany	1.1	AY259122	A	D	E	L	G	N	D	N	S	V	T	N	S	T	R	S	D	M	T	N
LPC	China	1.1	AY526732	A	D	E	L	G	T	D	N	T	V	T	N	S	T	G	F	D	M	T	N
GXBB1	China	2.1	AY450272	T	N	E	P	E	N	G	D	T	T	I	S	V	I	G	F	E	V	I	E
83-s106	China	2.1	AY526727	S	N	E	P	E	N	G	D	I	I	T	S	A	I	G	F	E	V	I	E
Paderborn	Germany	2.1	AY027673	S	N	E	P	E	N	G	D	I	T	T	S	A	I	G	F	E	V	I	E
GXNN1	China	2.1	AY450278	S	N	E	P	E	N	G	D	I	T	T	T	V	I	G	F	E	V	I	G
HZ2-04	China	2.1	EF683609	S	N	E	P	E	S	G	D	I	T	I	S	A	I	G	F	E	V	I	K
HZ-05	China	2.1	EF683629	S	N	E	P	E	S	G	D	I	T	I	R	A	I	G	F	E	V	M	K
HZ1-06	China	2.1	EF683626	S	N	E	P	E	S	G	D	I	T	I	S	A	I	G	F	E	V	I	K
QZ1-06	China	2.1	EF683618	S	N	E	P	E	S	G	D	I	T	I	S	A	I	G	F	E	V	I	K
QZ2-06	China	2.1	EF683619	S	N	E	P	E	S	G	D	I	T	I	S	A	I	G	F	E	V	I	K
HZ1-07	China	2.1	EF683627	S	N	E	P	E	S	G	D	I	T	M	S	A	I	G	F	E	V	I	K
QZ-07	China	2.1	FJ456876	S	N	E	P	E	N	G	D	I	T	I	S	A	I	G	F	E	V	V	K
HZ1-08	China	2.1	FJ582642	S	N	K	P	E	S	G	D	I	T	I	S	A	I	G	F	E	V	I	K
GXWZ02	China	2.1	AY367767	S	N	E	P	E	N	G	D	I	T	I	S	A	I	G	F	E	A	I	K
GX-HP3	China	2.1	AY450276	S	N	E	P	E	N	G	D	I	T	I	S	A	I	G	F	E	V	I	E
GS-ZY	China	2.1	AY450276	S	D	E	L	E	N	G	D	I	T	T	S	A	I	G	F	E	V	I	K
GS-HY	China	2.2	AF143086	A	N	E	L	E	S	D	D	A	I	T	S	V	I	G	F	E	V	M	K
HuB-39	China	2.2	AF407339	A	D	E	L	E	S	G	N	I	I	T	S	V	I	G	F	E	V	M	K
Mathura	India	2.2	EU567077	A	D	G	L	E	S	G	N	I	I	T	S	V	T	G	F	E	V	M	K
84-KS1	China	2.2	AY526729	A	N	E	L	E	S	G	D	I	I	T	S	V	I	G	F	E	V	M	K
LN1.84	China	2.2	DQ907717	A	N	E	L	E	G	G	D	I	I	T	S	V	I	G	F	E	V	M	K
Sukohario	Indonesia	2.2	EU180068	A	N	E	L	E	S	G	D	I	T	T	S	V	I	G	F	E	V	M	K
Roesrath	Germany	2.3	GU233734	A	N	E	L	E	S	D	D	V	T	T	N	V	I	G	F	D	V	I	K
Sp01	Spain	2.3	FJ265020	A	N	E	L	E	S	G	D	V	T	T	N	A	I	G	F	D	V	I	E
Alfor/T	Germany	2.3	J04358	A	N	E	L	E	S	G	D	V	T	T	N	A	I	G	F	D	V	I	K
Uelzen	Germany	2.3	GU324242	A	N	E	L	E	S	S	D	V	T	T	N	A	I	G	F	D	V	I	K
Substitutions based on C-strain rE2 protein by site-directed mutagenesis in this study				A→S	D→N	E→K	L→P	G→E	N→S	D→G	N→D	S→I	V→T	T→I	N→S	S→A	T→I	R→G	S→F	D→E	M→V	T→I	N→K
Enhancement of variant C-strain rE2 proteins in binding to the pig anti-QZ-07 serum^c^				-	++	-	++	++	++	+	+	-	-	-	+	++	+	-	-	+	+	+	+

^a^The five subgroup 1.1 strains listed in this table are the vaccine strains used in different countries. Twenty five representative group 2 strains are listed.

^b^Locations are derived from the polyprotein of classical swine fever virus C-strain (GenBank accession no. HM175885).

^c^Values represent binding efficiency of anti-QZ-07 serum to each of variant C-strain rE2 proteins: "++"= changes in binding of greater than 200% compared to that of wild type rE2 protein of C-strain; "+"= changes ranging from 150% to 200%; "-"= changes between 50% and 150%.

**Figure 5:**
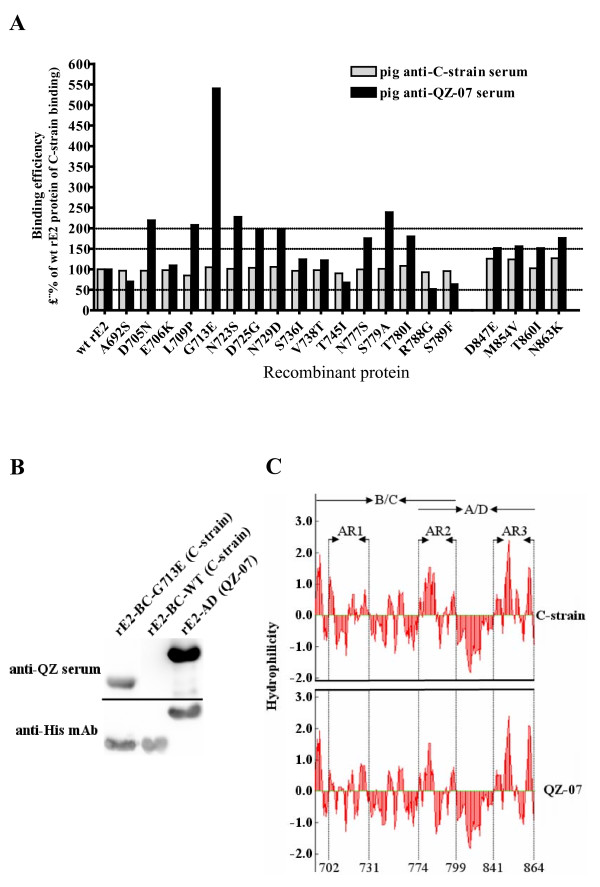
**Identification of residues and regions involved in antigenic variation of glycoprotein E2**. (A) Binding of the wild type (wt) and variant C-strain rE2 proteins to pig anti-C-strain or anti-QZ-07 sera. Site-directed mutagenesis was used to systematically substitute amino acids in C-strain E2 protein with those found at the same positions in subgroup 2.1 proteins. The substituted amino acids are depicted on the *x *axis. The *y *axis shows relative binding efficiency of individual rE2 proteins. For each of the variant C-strain rE2 proteins, the binding efficiency was determined by normalizing to anti-his-tag binding first, and then to the wt C-strain rE2 protein binding to pig anti-C-strain and anti-QZ-07 sera, respectively. Thus, the binding of the wt C-strain rE2 protein to either of the sera was set at 100%. rE2-BC proteins were used for A692S, D705N, E706K, L709P, G713E, N723S, D725G, N729D, S736I, V738T, T745I, N777S, S779A, T780I, R788G, and S789F substitutions because these residues are located in the antigenic unit B/C. rE2-AD proteins were used for D847E, M854V, T860I, and N863K substitutions since these residues are located in the antigenic unit A/D. The binding efficiency is relative to C-strain rE2-BC or rE2-AD binding to the reference serum depending on the kind of variant protein being compared. (B) Western blots of G713E variant rE2-BC protein using pig anti-QZ-07 serum and mouse monoclonal anti-His-tag antibody. The wt rE2-BC of C-strain and rE2-AD of strain QZ-07 are set as controls. (C) Hydrophilicity profile comparison of the antigenic units of E2 between the C-strain and strain QZ-07. The vertical axis represents the hydrophilicity scores.

We used site-directed mutagenesis to systematically substitute amino acids in C-strain E2 protein with those found at the same positions in subgroup 2.1 proteins (Table 3 - 2^nd ^last row). The binding of the wild type and variant C-strain rE2 proteins to C-strain and strain QZ-07 antisera was determined by binding ELISA. Wells of plates were coated with equal quantities of proteins and the antibodies were above saturation levels to ensure that antibody concentration was not limiting. The binding of the wt C-strain rE2 protein to either of the sera was set at 100%. None of the substitutions changed the binding of the variant rE2 proteins to anti-C-strain serum significantly (binding efficiency was between 80%-130%), suggesting that these residues did not contribute individually to the overall capacity of C-strain rE2 protein to bind the antibodies (Figure 5A). However, thirteen substitutions increased binding of the variant C-strain rE2 proteins to anti-QZ-07 serum (i.e., above 150% binding efficiency threshold). Substitution of D705N, L709P, G713E, N723S, or S779A caused a significant increase in binding efficiency (i.e., above 200% threshold), while a moderate increase was observed with D725G, N729D, N777S, T780I, D847E, M854V, T860I, or N863K substitution (between 150% and 200% efficiency). Remarkably, the G713E substitution dramatically enhanced binding of the variant rE2 protein to anti-QZ-07 serum as indicated by the more than 5-fold increase in binding efficiency (Figure 5A) and a strong reaction observed in the Western blot (Figure 5B). This residue is conserved within group 2 strains but different from the vaccine strains (Table 3), implying its role as a major determinant of antigenic variation.

The residues that caused significant or moderate increase of binding efficiency formed three distinct clusters in the antigenic units (Figure 1A). The first cluster is located in the N-terminus of antigenic unit B/C at the amino acid positions 702-731. The second cluster is at the boundary between the two antigenic units at positions 774-799 and the third one is in the C-terminus of antigenic unit A/D at positions 841-864. Interestingly, hydrophilicity analysis further demonstrated that these regions contribute to major hydrophilic differences between CSFV C-strain and strain QZ-07 (Figure 5C).

### Analysis of codon and amino acid diversity in the antigenic units of E2

**Figure 6:**
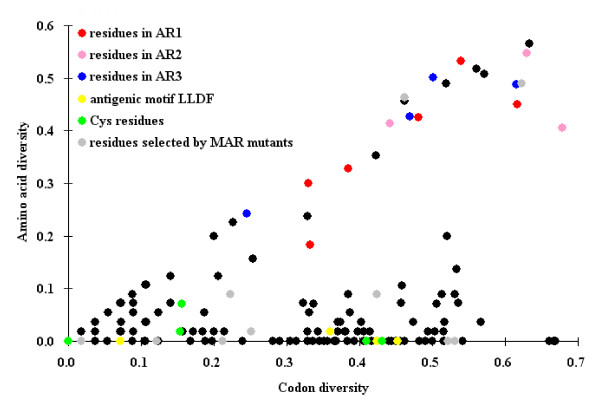
**Analysis of codon and amino acid diversity of residues within the antigenic units of glycoprotein E2**. Codon and amino acid diversity was quantified using a modified Simpson's index [41]. Antigenic residues identified in this study are colored according to the antigenic regions (AR) where they occur. Residues of the antigenic motif of ^771^LLFD^774 ^[25], the six conserved cysteine residues and the antigenic residues identified by mAb-resistant (MAR) mutants analysis [22], are marked in yellow, green and grey, respectively. The other residues are shown in black.

To get more insight into antigenic and genetic evolution of the antigenic units, the diversity of codon and amino acid was analyzed by a variant Simpson's index [41]. Figure 6 shows that the thirteen residues associated with antigenic variation (Figure 5 and Table 3) lie along the diagonal (*x = y*), indicating that these residues are highly diversified due to accumulation of large numbers of nonsynonymous mutations in their codons. In contrast, the six cysteine residues and residues in the ^771^LLFD^774 ^motif [25] lie along the *x *axis due to high conservation even though their codons have accumulated a moderate number of synonymous mutations. However, the antigenic residues identified by mAb-resistant mutants analysis [22] were mapped as having random distribution (Figure 6).

## Discussion

Phylogenetically, CSFV consists of three major groups [4]. Recent studies revealed that viral populations have shifted from the historical group 1 or 3 to group 2 in most European and Asian countries [4-10]. Glycoprotein E2 is a principal target of neutralizing antibodies and an important protective immunogen [16-21]. The E2 glycoproteins of three groups are genetically and antigenically different [4,10,11,25-35]. However, the basis of this antigenic variation has not been clearly demonstrated at the molecular level.

Our data show that both pig anti-C-strain and anti-QZ-07 sera bound heterologous rE2-AD proteins (from CSFV strain QZ-07 and C-strain, respectively) with <60% efficiency compared to homologous proteins (Figure 3A), indicating that these proteins are antigenically different. Further, the E2 protein of vaccine C-strain is antigenically distinct from those of a wide spectrum of subgroup 2.1 strains. Antigenic variation was also detected among subgroup 2.1 strains as indicated by the inefficiency of pig anti-QZ-07 serum to bind HZ1-08- and QZ2-06-derived rE2-AD proteins (Figure 3). Our data further demonstrate that the previously reported differences in antigenicity detected by mouse mAbs [11,25,30-35] also occur in the context of pig anti-CSFV sera.

We performed neutralization experiments to assess whether the differences in the efficiency of antibody binding to rE2 proteins (Figure 3) correlate with the ability of the antibody to block CSFV infection. A two-way neutralization determination showed that pig anti-CSFV sera neutralized heterologous strains less efficiently (Figure 4). Rabbit polyclonal antibodies against purified C-strain rE2-AD protein also showed less efficiency at neutralizing strain QZ-07. Furthermore, two conformational anti-C-strain-rE2-AD mAbs (1E7 and 6B8) had lower binding and neutralization efficiency against the heterologous strains compared to C-strain (Table 2), suggesting that the neutralization differences seen with pig anti-CSFV sera were, at least in part, due to differential expression of antigenic epitopes on the E2 glycoproteins of CSFV strains. Such antigenic variation may explain why subgroup 2.1 CSFV strains persist in China despite the wide use of vaccine C-strain. Antibody selection may be one of the reasons for the switch of viral populations from group 1 to 2.

We used site-directed mutagenesis to introduce amino acid substitutions in the C-strain rE2 proteins in order to probe whether variable residues (Table 3) contribute to the antigenic variation seen with subgroup 2.1 strains. Unlike the mutations in the antigenic motif ^771^LLFD^774 ^that disrupted the structural integrity of E2 protein [25], none of the substitutions had a significant effect on binding to anti-C-strain serum (Figure 5A). We infer that the recombinant proteins were not grossly misfolded and the substituted residues may not be critical for the overall structural stability of glycoprotein E2. In contrast, of the 20 substitutions, 13 enhanced binding of the variant C-strain rE2 proteins to anti-QZ-07 serum (Figure 5A). The most dramatic increase in binding was caused by the G→E substitution at aa position 713 (Figure 5A and 5B). Sequence alignment revealed that all group 2 strains have residue ^713^E, while all the vaccine strains have ^713^G (Table 3). Chang et al. recently reported that residues ^713^E and ^729^D were critical for specificity of a group 3.4 field strain rE2 protein to mAbs [35]. It appears that ^713^E is a common antigenic determinant for both groups 2 and 3. Our work demonstrates that although residue ^729^D enhanced binding to pig anti-QZ-07 serum, residues ^705^N, ^709^P, ^723^S, and ^779^A had much more significant contribution (Figure 5A). Notably, the same residues are found at positions 705 and 723 on E2 proteins of subgroup 2.1 and subgroup 3.4 strains. It is possible that these two residues may also show superior contribution to the antigenicity of subgroup 3.4 glycoprotein E2 if probed with pig antisera against group 3 strains. In this study, we used polyclonal sera from pigs C-strain-immunized or infected with a field strain which contained the full spectrum of immunization- or infection-induced antibodies. This is why these polyclonal sera could identify more residues responsible for antigenic variation of glycoprotein E2 than mouse mAbs [35]. Furthermore, pairing of the polyclonal antisera against the group 1 C-strain and representative group 2 field strain could probe the residues that mediate antigenic variation between the two groups, another advantage over mAbs.

Based on the data revealed by the site-directed mutagenesis analysis (Figure 5A), the antigenic variation among subgroup 2.1 strains is not unexpected since each of the 8 subgroup 2.1 strains used in this study has some unique strain-specific substitutions (data not shown). The C737R substitution in the antigenic units of strain QZ2-06 appears to affect binding the most. This can be explained by the fact that the cysteine residue at this position is critical for the antigenic structure of the protein [22]. We speculate that E782V substitution in strain HZ1-08 is the key determinant of antigenic variation between strain HZ1-08 and our reference subgroup 2.1 strain QZ-07.

Three discrete antigenic regions were mapped at aa positions 702-731, 774-799 and 841-864, in the antigenic units of E2 protein (Figure 1A). Several antigenic residues identified by mAb-resistant mutants analysis [22] or epitope mapping [35] and substitutions with significant increase in binding of variant rE2 proteins to anti-QZ-07 serum examined in this study are clustered in the 702-731 region (Figure 5A), implying that evolution of this region is the primary cause of antigenic variation of glycoprotein E2. The N-terminus of antigenic region 774-799 contains the conserved antigenic motif ^771^LLFD^774 ^[25] and a conserved linear ^772^LFDGTNP^778 ^epitope [39], suggesting its essential role in maintaining the integrity of antigenic structure of E2 protein. In addition, the substitutions of N777S, S779A, and T780I in this region enhanced binding of variant rE2 proteins to anti-QZ-07 serum (Figure 5A). Therefore, region 774-799 may have multiple functions in shaping the antigenicity of E2.

Finally, we analyzed E2 sequences of CSFV in order to compare codon and amino acid diversification in relation to antigenic evolution. We employed a variant Simpson's index that has been used to quantify codon and amino acid diversity in the antigenic epitopes of influenza virus hemagglutinin glycoprotein [41,42]. The diversity of each of the thirteen amino acid residues involved in antigenic variation is equivalent to that of the corresponding codon (Figure 6: the unique distribution along the *x *= *y *diagonal), indicating a remarkable correlation between genetic and antigenic evolution within the antigenic units of glycoprotein E2 in nature. In contrast, the antigenic residues identified by mAb-resistant mutants analysis [22] are randomly diversified (Figure 6: randomly distributed grey-colored residues), suggesting that in vitro selection may not explain natural selection in pig. Co-diversification of codons and amino acids involved in antigenic variation in the field strains could be one of the immune evasion mechanisms that CSFV employs under immune pressure as a result of extensive vaccination [43].

## Conclusions

This study demonstrates antigenic variation of CSFV glycoprotein E2 between the vaccine C-strain and group 2 field strains or even within group 2 strains currently circulating in China. Of the three discrete regions associated with antigenic variation, substitutions in the first region (aa 702-731) are the primary determinants of the antigenic variation of E2. Since glycoprotein E2 variation affects CSFV cross-neutralization, subsequent work will determine whether these antigenic residues contribute to the observed neutralization differences. Our findings may provide useful information for the development of differential serological assays and novel CSF vaccines with improved immunogenicity and efficacy.

## Materials and Methods

### Cells and viruses

Swine testicle (ST) cells were grown in Minimum Essential Medium (MEM, Gibco, USA) supplemented with 10% fetal bovine serum (FBS). The following CSFV strains were used: the subgroup 1.1 vaccine C-strain widely used for prophylactic vaccination in China and two subgroup 2.1 strains recently circulating in China (strains QZ-07 and HZ1-08). CSFV vaccine C-strain was obtained from Zhejiang Jianliang Biological Engineering Company (Zhejiang province, China). Two subgroup 2.1 strains were originally isolated from spleens of naturally infected pigs and replicated in ST cells in our laboratory. These three viruses were propagated and titrated in ST cells. Stocks were aliquoted and stored at -80°C until use. The virus stocks were sequenced to confirm that the E2 genes had the expected sequences. The other 6 subgroup 2.1 strains were not isolated and only their E2 genes were directly cloned in plasmids. Sequence data is available in GenBank as listed in Table 3. Details of their molecular phylogenetic relationships have been described elsewhere [10,26].

### E2 sequence dataset

All E2 sequences covering the complete antigenic region were retrieved from NCBI database. The nucleotide and amino acid sequences were aligned using Clustal X software (version 1.83). Sequences with 100% nucleotide identity were excluded. The remaining sequences included 23, 82 and 3 sequences representing groups 1, 2 and 3, respectively. This dataset was used to identify the major variable residues (see Table 3) and to analyze the codon and amino acid diversity (Figure 6).

### Construction of expression plasmids

The plasmids containing full-length E2 gene of the vaccine C-strain and eight subgroup 2.1 strains used in this study were previously described [10,26]. The C-strain specific primer sets C-E2-AD-f/C-E2-AD-r and C-E2-BC-f/C-E2-BC-r were used to amplify the fragment covering the two antigenic units (B/C+A/D) and the fragment only containing antigenic unit B/C, respectively. Primer set QZ-E2-AD-f/QZ-E2-AD-r was used to amplify the fragments covering the two antigenic units of group 2 isolates (Table 1). PCR amplicons were digested with restriction enzymes *Bam*HI and *Xho*I, gel purified and ligated into prokaryotic expression vector pET-30a(+). To construct the eukaryotic expression plasmid, a 1212-bp cDNA fragment encoding the signal sequence and full-length E2 of C-strain was amplified with primer set C-E2-f and C-E2-r (Table 1), and cloned into pcDNA3.1 following *Bam*HI and *Xho*I digestion.

### Expression and purification of the prokaryotic-derived, His-tagged rE2 proteins

*E. coli *Rosetta (DE3) cells containing different recombinant plasmids were cultured to an optical density (OD) between 0.6 and 0.8 at 600 nm. Expression of His-tagged rE2 proteins was induced with 1 mM isopropyl-β-D-thiogalactoside (IPTG, Sigma-Aldrich). Cells were harvested and disrupted by sonication. After centrifugation, the inclusion bodies with rE2 proteins were resuspended with 1/10 volume of buffer (100 mM NaH_2_PO_4_·2H_2_O, 10 mM Tris-base, and 8 M Urea). The supernatant was collected after centrifugation and purified by Ni-NTA affinity column (Novagen, Madison, WI) according to the manufacturer's protocol. Finally, the proteins were refolded by washing the column with 40 ml of Tris-buffered saline (TBS, pH 7.4) containing 1 M urea and eluted from the column with 200 mM imidazole in TBS. The purified rE2 proteins were confirmed by Western blotting with mouse monoclonal anti-His-tag antibody (Sigma-Aldrich) and quantified by the Bradford assay.

### Production of antibodies against CSFV C-strain and strain QZ-07

The pig hyperimmune serum against CSFV vaccine C-strain was previously prepared and stocked in our laboratory. The pig antiserum to the C-strain (pig anti-C-strain) or to the strain QZ-07 (pig anti-QZ-07) was induced by intramuscular immunization of 30-day-old CSFV-free pigs with the attenuated vaccine C-strain by prime-boost strategy or infection with 10^5 ^TCID_50 _of strain QZ-07 in a biosafety level III facility, respectively. The sera were collected at different times post-vaccination or infection and stored at -80°C until use. The sera at highest titers collected at 78 days post immunization with the C-strain and 25 days post infection with strain QZ-07 (see Figure 2) were used for binding ELISAs in Figure 3A and Figure 5A and Western blots in Figure 3B and Figure 5B.

The rabbit antiserum to the rE2-AD protein of C-strain was generated as follows: New Zealand white rabbits were immunized and boosted two times with 0.5 mg of the purified rE2-AD protein of C-strain (expressed in *E. coli*) emulsified with an equal volume of complete/incomplete Freund's adjuvant (Sigma-Aldrich). Blood was drawn for antiserum preparation once maximum level of antibody production was reached.

For monoclonal antibodies against the rE2-AD protein of C-strain, four 5-week-old female specific-pathogen-free BALB/c mice were immunized subcutaneously with 0.1 mg of the purified rE2-AD protein of vaccine C-strain emulsified in complete Freund's adjuvant. The mice were intraperitoneally boosted twice with rE2-AD protein emulsified in incomplete Freund's adjuvant at 2-week intervals. The mice were euthanized 2 weeks after the last boosting and spleen cells were harvested. Splenocytes were fused with SP2/0 myeloma cells using 50% (v/v) polyethylene glycol (PEG, Sigma-Aldrich). The resulting hybridomas secreting antibodies against rE2-AD protein were selected by immunofluorescence assay (IFA), and then clonally expanded. Antibody subtyping was performed using mouse mAb Isotyping Reagents (Sigma-Aldrich) according to the manufacturer's instructions. Ascites were produced in pristine-primed BALB/c mice. Experiments with animals were approved by the Laboratory Animal Management Committee (animal welfare ethics is part of its duties) of Zhejiang University.

### Site-directed mutagenesis of the C-strain based E2 proteins

To identify the antigenic units recognized by mAbs, cysteine codons of the C-strain E2 gene in eukaryotic expression plasmid were mutated to serine codons by site-directed mutagenesis as described previously [22].

Multiple E2 sequence alignment was used to identify variable residues. Twenty major variable residues were identified in the antigenic units (Table 3). These do not include K→R or S→T substitutions (K720R, K734R, K761R, S797T, and R845K substitutions). To substitute C-strain residues for those found in group 2 isolates, plasmids encoding individual mutations (listed in Table 3) were generated by site-directed mutagenesis. Substitutions were made on plasmids encoding the antigenic unit B/C or two units (B/C+A/D) of C-strain E2 protein depending on where the residue being substituted is located in the antigenic units.

All substitutions were performed using QuikChange Site-Directed Mutagenesis Kit (Stratagene CA, USA) according to the manufacturer's instructions. The primers were designed via the QuikChange Primer Design Program http://www.stratagene.com. The desired nucleotide changes in each mutant were verified by sequencing. Expression and purification of variant rE2 proteins was done as mentioned above.

### Binding ELISA with recombinant E2 proteins

All ELISAs described in this study were performed in triplicate under stringent conditions to avoid nonspecific reactions. Antibodies were diluted using phosphate-buffered saline (PBS, pH 7.4) containing 5% nonfat dry milk (PBS/NFDM); each washing step included 5 washes with PBS containing 0.5% Tween 20 (PBS/Tween). Briefly, a 100-μl volume of different rE2 proteins (10 μg/ml in 50 mM sodium carbonate buffer, pH 9.6) was added into each well of 96-well microtiter plates (MaxiSorp, Nunc, Denmark) for overnight incubation at 4°C. The wells were washed with PBS/Tween and then blocked with PBS/NFDM at 37°C for 2 h. The wells were washed and incubated with different antibodies for 1 h. The wells were washed again and then incubated with horseradish peroxidase conjugated SPA at 37°C for 1 h. Thereafter, wells were washed and incubated with 100 μl/well of the chromogenic substrate 3,3',5,5'-tetramethylbenzidine (TMB, Sigma-Aldrich) at 37°C for 4 min. The reaction was stopped by adding 50 μl of 2 M H_2_SO_4_. Finally, the OD_450 nm _was measured using spectraMax^@^M2 microplate reader (Molecular devices Corp., USA).

The binding efficiency of rE2-AD proteins from C-strain and 8 subgroup 2.1 strains with the two pig antisera to the C-strain and strain QZ-07 (Figure 3A) was normalized to anti-His-tag binding first, and then expressed as the ratio of antibody bound to individual group 2 rE2-AD protein to that bound to the rE2-AD proteins of C-strain or strain QZ-07, which was set at 100%. The mean binding efficiency of each individual protein was calculated for three independent ELISA assays.

For variant C-strain rE2 proteins in Figure 5A, rE2-BC proteins were used for A692S, D705N, E706K, L709P, G713E, N723S, D725G, N729D, S736I, V738T, T745I, N777S, S779A, T780I, R788G, and S789F substitutions because these residues are located in the antigenic unit B/C. rE2-AD proteins were used for D847E, M854V, T860I, and N863K substitutions since these residues are located in the antigenic unit A/D. The results were first normalized to anti-His-tag binding and then expressed as the ratio of their binding to the antibodies to that of binding to C-strain wild type rE2-BC or rE2-AD binding to the reference serum depending on the kind of variant protein being compared. Relative binding of greater than 200% efficiency were designated as significant increases in antibody binding. Binding efficiencies between 150% and 200% efficiency were considered as moderate increases whereas those between 50% and 150% efficiency were considered as limited effect on antibody binding.

### Western blot analysis

The antigenic reactivity of different rE2 proteins was assessed by Western blotting. The proteins were separated by 15% SDS-PAGE and transferred to nitrocellulose membranes (PALL Corp., USA). The membranes were subsequently blocked (overnight at 4°C) in blocking buffer (PBS/NFDM) and then incubated at 37°C for 1 h with different antibodies. After incubation, membranes were rinsed for 20 min in PBS/Tween, and bound antibodies were detected with SPA-conjugated with horseradish peroxidase diluted at 1:2500. For color development, 4-chloro-1-naphthol (4-CN, Sigma-Aldrich) was used.

### Virus neutralization assay

The neutralization indices (NI) of the antibodies against different CSFV strains were determined by virus neutralization assay. Briefly, ST cells were seeded in 96-well tissue culture plates and incubated overnight at 37°C. Two-fold serial dilutions of the different heat-inactivated sera were mixed with equal volumes of 100 TCID_50 _virus suspensions, incubated at 37°C for 1 h and subsequently transferred to confluent monolayers of ST cells in 96-well plates. The starting dilution of each serum was 1:50. At 72 hours post-infection, the cells were fixed and stained for the presence of glycoprotein E2 by immunofluorescence assay. The NI is the log_10 _of the antibody dilution factor (reciprocal of dilution) when 50% of the wells are protected from infection. Since the starting dilution factor was 50, the NI value of 1.7 is the detection threshold of our neutralization assay.

### Immunofluorescence assay

Immunofluorescence assay (IFA) was used to verify the reactivity of the CSFV strains or cysteine-mutated E2 proteins with different antibodies. Briefly, cells infected with CSFV strains at 72 h or cells transfected with cysteine-mutated recombinant plasmids at 48 h were fixed in 3.7% paraformaldehyde at room temperature for 60 min and permeabilized for 10 min with 0.1% Triton X-100 in PBS. The cells were incubated for 1 h with different antibodies, and then stained with goat anti-rabbit antibody conjugated with Texas green or goat anti-mouse antibody conjugated with Alexa red (Molecular Probes Inc., USA) for another 1 h. Cells were examined under the IX71 inverted fluorescence microscope (Olympus, Japan).

### Hydrophobicity profile and evolution analysis within antigenic units of E2

Hydrophobicity profile was generated using DNASIS software by the method of Kyte and Doolittle [44]. Evolution analysis was performed using an information-theoretic method described by Plotkin and Dushoff [41]. Briefly, we plotted the diversity of codons found at each residue against the diversity of amino acids found at the same residue. The diversity of codons or amino acids was quantified by a variant Simpson's index: D = 1-p_*i*_^2^, where p_i _denotes the relative frequency of the *i*-th codon or amino acid at the residue in the multiple sequence alignment.

## Competing interests

The authors declare that they have no competing interests.

## Authors' contributions

NC conceived and designed the study, carried out the plasmids construction and site-directed mutagenesis, performed data analysis and drafted the manuscript. CT contributed to the neutralization assay and participated in sequence analysis. DL expressed and purified the proteins used in this study, performed Western blot and ELISA analysis. JW, XY and XL produced the antibodies and participated in sequence analysis. JP contributed to the experimental design and provided critical review of the manuscript. WF supervised the project, participated in the design of the study and data interpretation, and helped draft the manuscript. All authors read and approved the final manuscript.

## References

[B1] Moennig V Introduction to classical swine fever: virus, disease and control policy Vet Microbiol 2000 73 93 102 10.1016/S0378-1135(00)00137-1 10785320

[B2] Becher P Avalos Ramirez R Orlich M Cedillo Rosales S Konig M Schweizer M Stalder H Schirrmeier H Thiel HJ Genetic and antigenic characterization of novel pestivirus genotypes: implications for classification Virology 2003 311 96 104 10.1016/S0042-6822(03)00192-2 12832207

[B3] Liu L Xia H Wahlberg N Belak S Baule C Phylogeny, classification and evolutionary insights into pestiviruses Virology 2009 385 351 357 10.1016/j.virol.2008.12.004 19167739

[B4] Paton DJ McGoldrick A Greiser-Wilke I Parchariyanon S Song JY Liou PP Stadejek T Lowings JP Bjorklund H Belak S Genetic typing of classical swine fever virus Vet Microbiol 2000 73 137 157 10.1016/S0378-1135(00)00141-3 10785324

[B5] Bartak P Greiser-Wilke I Genetic typing of classical swine fever virus isolates from the territory of the Czech Republic Vet Microbiol 2000 77 59 70 10.1016/S0378-1135(00)00260-1 11042400

[B6] Cha SH Choi EJ Park JH Yoon SR Kwon JH Yoon KJ Song JY Phylogenetic characterization of classical swine fever viruses isolated in Korea between 1988 and 2003 Virus Res 2007 126 256 261 10.1016/j.virusres.2007.01.017 17328983

[B7] Deng MC Huang CC Huang TS Chang CY Lin YJ Chien MS Jong MH Phylogenetic analysis of classical swine fever virus isolated from Taiwan Vet Microbiol 2005 106 187 193 10.1016/j.vetmic.2004.12.014 15778024

[B8] Greiser-Wilke I Fritzemeier J Koenen F Vanderhallen H Rutili D De Mia GM Romero L Rosell R Sanchez-Vizcaino JM San Gabriel A Molecular epidemiology of a large classical swine fever epidemic in the European Union in 1997-1998 Vet Microbiol 2000 77 17 27 10.1016/S0378-1135(00)00253-4 11042397

[B9] Tu C Lu Z Li H Yu X Liu X Li Y Zhang H Yin Z Phylogenetic comparison of classical swine fever virus in China Virus Res 2001 81 29 37 10.1016/S0168-1702(01)00366-5 11682122

[B10] Chen N Hu H Zhang Z Shuai J Jiang L Fang W Genetic diversity of the envelope glycoprotein E2 of classical swine fever virus: recent isolates branched away from historical and vaccine strains Vet Microbiol 2008 127 286 299 10.1016/j.vetmic.2007.09.009 17976931

[B11] Zhu Y Shi Z Drew TW Wang Q Qiu H Guo H Tu C Antigenic differentiation of classical swine fever viruses in China by monoclonal antibodies Virus Res 2009 142 169 174 10.1016/j.virusres.2009.02.011 19428750

[B12] Reimann I Depner K Trapp S Beer M An avirulent chimeric Pestivirus with altered cell tropism protects pigs against lethal infection with classical swine fever virus Virology 2004 322 143 157 10.1016/j.virol.2004.01.028 15063124

[B13] Wang Z Nie Y Wang P Ding M Deng H Characterization of classical swine fever virus entry by using pseudotyped viruses: E1 and E2 are sufficient to mediate viral entry Virology 2004 330 332 341 10.1016/j.virol.2004.09.023 15527858

[B14] Risatti GR Borca MV Kutish GF Lu Z Holinka LG French RA Tulman ER Rock DL The E2 glycoprotein of classical swine fever virus is a virulence determinant in swine J Virol 2005 79 3787 3796 10.1128/JVI.79.6.3787-3796.2005 15731272 PMC1075681

[B15] Risatti GR Holinka LG Fernandez Sainz I Carrillo C Kutish GF Lu Z Zhu J Rock DL Borca MV Mutations in the carboxyl terminal region of E2 glycoprotein of classical swine fever virus are responsible for viral attenuation in swine Virology 2007 364 371 382 10.1016/j.virol.2007.02.025 17418362

[B16] Beer M Reimann I Hoffmann B Depner K Novel marker vaccines against classical swine fever Vaccine 2007 25 5665 5670 10.1016/j.vaccine.2006.12.036 17239502

[B17] Bouma A de Smit AJ de Kluijver EP Terpstra C Moormann RJ Efficacy and stability of a subunit vaccine based on glycoprotein E2 of classical swine fever virus Vet Microbiol 1999 66 101 114 10.1016/S0378-1135(99)00003-6 10227472

[B18] de Smit AJ Bouma A de Kluijver EP Terpstra C Moormann RJ Duration of the protection of an E2 subunit marker vaccine against classical swine fever after a single vaccination Vet Microbiol 2001 78 307 317 10.1016/S0378-1135(00)00306-0 11182497

[B19] Hulst MM Westra DF Wensvoort G Moormann RJ Glycoprotein E1 of hog cholera virus expressed in insect cells protects swine from hog cholera J Virol 1993 67 5435 5442 8350404 10.1128/jvi.67.9.5435-5442.1993 PMC237945

[B20] van Rijn PA Bossers A Wensvoort G Moormann RJ Classical swine fever virus (CSFV) envelope glycoprotein E2 containing one structural antigenic unit protects pigs from lethal CSFV challenge J Gen Virol 1996 77 Pt 11 2737 2745 10.1099/0022-1317-77-11-2737 8922467

[B21] Weiland E Stark R Haas B Rumenapf T Meyers G Thiel HJ Pestivirus glycoprotein which induces neutralizing antibodies forms part of a disulfide-linked heterodimer J Virol 1990 64 3563 3569 2370675 10.1128/jvi.64.8.3563-3569.1990 PMC249648

[B22] van Rijn PA Miedema GK Wensvoort G van Gennip HG Moormann RJ Antigenic structure of envelope glycoprotein E1 of hog cholera virus J Virol 1994 68 3934 3942 7514680 10.1128/jvi.68.6.3934-3942.1994 PMC236899

[B23] van Rijn PA van Gennip HG de Meijer EJ Moormann RJ Epitope mapping of envelope glycoprotein E1 of hog cholera virus strain Brescia J Gen Virol 1993 74 Pt 10 2053 2060 10.1099/0022-1317-74-10-2053 7691986

[B24] Lin M Lin F Mallory M Clavijo A Deletions of structural glycoprotein E2 of classical swine fever virus strain alfort/187 resolve a linear epitope of monoclonal antibody WH303 and the minimal N-terminal domain essential for binding immunoglobulin G antibodies of a pig hyperimmune serum J Virol 2000 74 11619 11625 10.1128/JVI.74.24.11619-11625.2000 11090160 PMC112443

[B25] Chang CY Huang CC Lin YJ Deng MC Chen HC Tsai CH Chang WM Wang FI Antigenic domains analysis of classical swine fever virus E2 glycoprotein by mutagenesis and conformation-dependent monoclonal antibodies Virus Res 2010 149 183 189 10.1016/j.virusres.2010.01.016 20132851

[B26] Chen N Li D Yuan X Li X Hu H Zhu B Wan X Fang W Genetic characterization of E2 gene of classical swine fever virus by restriction fragment length polymorphism and phylogenetic analysis Virus Genes 2010 40 389 396 10.1007/s11262-010-0465-8 20217206

[B27] Lowings JP Paton DJ Sands JJ De Mia GM Rutili D Classical swine fever: genetic detection and analysis of differences between virus isolates J Gen Virol 1994 75 Pt 12 3461 3468 10.1099/0022-1317-75-12-3461 7996138

[B28] Parchariyanon S Inui K Damrongwatanapokin S Pinyochon W Lowings P Paton D Sequence analysis of E2 glycoprotein genes of classical swine fever viruses: identification of a novel genogroup in Thailand Dtsch Tierarztl Wochenschr 2000 107 236 238 10916939

[B29] Shiu JS Chang MH Liu ST Ho WC Lai SS Chang TJ Chang YS Molecular cloning and nucleotide sequence determination of three envelope genes of classical swine fever virus Taiwan isolate p97 Virus Res 1996 41 173 178 10.1016/0168-1702(96)01286-5 8738176

[B30] Edwards S Sands JJ Antigenic comparisons of hog cholera virus isolates from Europe, America and Asia using monoclonal antibodies Dtsch Tierarztl Wochenschr 1990 97 79 81 2178905

[B31] Kosmidou A Ahl R Thiel HJ Weiland E Differentiation of classical swine fever virus (CSFV) strains using monoclonal antibodies against structural glycoproteins Vet Microbiol 1995 47 111 118 10.1016/0378-1135(95)00054-E 8604543

[B32] Mendoza S Correa-Giron P Aguilera E Colmenares G Torres O Cruz T Romero A Hernandez-Baumgarten E Ciprian A Antigenic differentiation of classical swine fever vaccinal strain PAV-250 from other strains, including field strains from Mexico Vaccine 2007 25 7120 7124 10.1016/j.vaccine.2007.07.045 17728020

[B33] Nishimori T Yamada S Shimizu M Production of monoclonal antibodies against classical swine fever virus and their use for antigenic characterization of Japanese isolates J Vet Med Sci 1996 58 707 710 8844614 10.1292/jvms.58.707

[B34] van Rijn PA A common neutralizing epitope on envelope glycoprotein E2 of different pestiviruses: implications for improvement of vaccines and diagnostics for classical swine fever (CSF)? Vet Microbiol 2007 125 150 156 10.1016/j.vetmic.2007.05.001 17561359

[B35] Chang CY Huang CC Lin YJ Deng MC Tsai CH Chang WM Wang FI Identification of antigen-specific residues on E2 glycoprotein of classical swine fever virus Virus Res 2010 152 65 72 10.1016/j.virusres.2010.06.005 20558217

[B36] Clavijo A Lin M Riva J Mallory M Lin F Zhou EM Development of a competitive ELISA using a truncated E2 recombinant protein as antigen for detection of antibodies to classical swine fever virus Res Vet Sci 2001 70 1 7 10.1053/rvsc.2000.0434 11170845

[B37] Kesik M Saczynska V Szewczyk B Plucienniczak A Inclusion bodies from recombinant bacteria as a novel system for delivery of vaccine antigen by the oral route Immunol Lett 2004 91 197 204 10.1016/j.imlet.2003.12.001 15019290

[B38] Lin M Trottier E Mallory M Enzyme-linked immunosorbent assay based on a chimeric antigen bearing antigenic regions of structural proteins Erns and E2 for serodiagnosis of classical swine fever virus infection Clin Diagn Lab Immunol 2005 12 877 881 16002639 10.1128/CDLI.12.7.877-881.2005 PMC1182199

[B39] Peng WP Hou Q Xia ZH Chen D Li N Sun Y Qiu HJ Identification of a conserved linear B-cell epitope at the N-terminus of the E2 glycoprotein of Classical swine fever virus by phage-displayed random peptide library Virus Res 2008 135 267 272 10.1016/j.virusres.2008.04.003 18485511

[B40] Sung JH Kang ML Lee WJ Shin MK Lim SI Kim BH Song JY Yoo HS Improved sero-monitoring assay for classical swine fever (CSF) using the recombinant E2 protein of a recent Korean isolate Res Vet Sci 2010 20591455 10.1016/j.rvsc.2010.06.003

[B41] Plotkin JB Dushoff J Codon bias and frequency-dependent selection on the hemagglutinin epitopes of influenza A virus Proc Natl Acad Sci USA 2003 100 7152 7157 10.1073/pnas.1132114100 12748378 PMC165845

[B42] Li J Wang Y Liang Y Ni B Wan Y Liao Z Chan KH Yuen KY Fu X Shang X Fine antigenic variation within H5N1 influenza virus hemagglutinin's antigenic sites defined by yeast cell surface display Eur J Immunol 2009 39 3498 3510 10.1002/eji.200939532 19798682

[B43] Lipsitch M O'Hagan JJ Patterns of antigenic diversity and the mechanisms that maintain them J R Soc Interface 2007 4 787 802 10.1098/rsif.2007.0229 17426010 PMC2394542

[B44] Kyte J Doolittle RF A simple method for displaying the hydropathic character of a protein J Mol Biol 1982 157 105 132 10.1016/0022-2836(82)90515-0 7108955

